# Extraction of Preisach model parameters for fluorite-structure ferroelectrics and antiferroelectrics

**DOI:** 10.1038/s41598-021-91492-w

**Published:** 2021-06-14

**Authors:** Zheng Wang, Jae Hur, Nujhat Tasneem, Winston Chern, Shimeng Yu, Asif Khan

**Affiliations:** 1 Georgia Institute of Technology, School of Electrical and Computer Engineering, Atlanta, 30332 USA; 2 Department of Electrical Engineering and Computer Science, Massachusetts Institute of Technology, Cambridge, MA 02142 USA; 3 Izentis LLC, PO Box 397002, Cambridge, MA 02139 USA; 4 Georgia Institute of Technology, School of Materials Science and Engineering, Atlanta, 30332 USA

**Keywords:** Electrical and electronic engineering, Electronic devices

## Abstract

Flourite-structure ferroelectrics (FEs) and antiferroelectrics (AFEs) such as HfO_2_ and its variants have gained copious attention from the semiconductor community, because they enable complementary metal-oxide-semiconductor (CMOS)-compatible platforms for high-density, high-performance non-volatile and volatile memory technologies. While many individual experiments have been conducted to characterize and understand fluorite-structure FEs and AFEs, there has been little effort to aggregate the information needed to benchmark and provide insights into their properties. We present a fast and robust modeling framework that automatically fits the Preisach model to the experimental polarization ($Q_{FE}$) versus electric field ($E_{FE}$) hysteresis characterizations of fluorite-structure FEs. The modifications to the original Preisach model allow the double hysteresis loops in fluorite-structure antiferroelectrics to be captured as well. By fitting the measured data reported in the literature, we observe that ferroelectric polarization and dielectric constant decrease as the coercive field rises in general.

## Introduction

**Figure 1:**
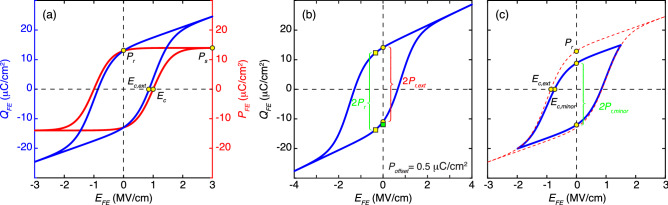
Issues with direct determination of ferroelectric parameters. (**a**) A typical ferroelectric hysteresis loop ($Q_{FE}$-$E_{FE}$), where $E_{c,ext}$ directly determined at $Q_{FE}$ = 0 μC/cm^2^ is smaller than the actually $E_{c}$ from the $P_{FE}$-$E_{FE}$ loops. In addition, $P_s$ is not obvious from the $Q_{FE}$-$E_{FE}$ curve. (**b**) A hysteresis loop with asymmetrical $E_{c\pm }$ and non-zero $P_{offset}$, where 2$P_{r,ext}$ is smaller than 2$P_r$, leading to underestimation. The green square indicates the endpoint of the 2$P_r$ when the start point (top yellow square) of the 2$P_r$ aligned with that (top yellow circle) of the 2$P_{r,ext}$. (**c**) A minor hysteresis loop (blue) and the corresponding saturated hysteresis loop (dashed red), where 2$P_{r,minor}$ is smaller than 2$P_r$ and $E_{c,minor}$ is less than $E_{c,ext}$. Extracting ferroelectric parameters from a minor loop leads to underestimation.

Recent rapid development in data-intensive computing necessitates novel memory technologies beyond traditional flash memory. Ferroelectric field-effect transistors (FeFETs), as emerging memory, find a niche in such applications due to their ultra-fast program/erase time, low operation voltage, and low power consumption^1–3^. Despite the fact that hafnium oxide^4,5^ and its doped variants (Al-doped^6,7^, Gd-doped^8^, La-doped^9^, Si-doped^1,10–12^, Sr-doped^13^, Y-doped^14,15^, Zr-doped^4,10,16–26^) have been extensively studied and characterized over the past few years, little has been done to aggregate those data into ferroelectric properties to provide the insight necessary to create a predictive model for ferroelectrics. Such a predictive model cannot be realized without the accurate determination of a multitude of ferroelectric parameters from various experimental hysteresis loops ($Q_{FE}$-$E_{FE}$). However, direct determination of ferroelectric parameters, such as polarization ($P_s$ and $P_r$) and coercive field ($E_c$), from ferroelectric hysteresis characteristics can be inaccurate. Conventionally, the remnant polarization $P_r$ is evaluated at $E_{FE}$ = 0 MV/cm, the coercive field $E_c$ is determined at $P_{FE}$ = 0 μC/cm^2^, and the saturated polarization $P_s$ is the maximum $P_{FE}$. Due to the contribution from the linear dielectric component of ferroelectrics ($\varepsilon _0\varepsilon _{FE}E_{FE}$), evaluating the coercive field $E_c$ at $Q_{FE}$ = 0 μC/cm^2^ directly would underestimate the actual coercive field and the value of $P_s$ is not obvious on the $Q_{FE}$-$E_{FE}$ hysteresis loop (Fig. 1a). In addition, non-ideal effects such as asymmetrical $E_{c\pm }$ and polarization offset $P_{offset}$ hinder the direct parameter identification (Fig. 1b). Furthermore, the actual hysteresis loop measured could be a non-saturated minor loop, thus extracting ferroelectric parameters from such a loop would lead to significant underestimation (Fig. 1c). In summary, direct determination of ferroelectric parameters from a single hysteresis loop, a technique ubiquitously used in the literature, is prone to undervaluing ferroelectric parameters and unable to accurately extract some ferroelectric parameters.

To overcome the problem, we present a fast and robust modeling framework that automatically fits experimental ferroelectric hysteresis loops based on a computationally efficient Preisach model that had been widely adopted to model hysteresis of ferroelectrics in the past^27^, and we further extend this model to describe antiferroelectrics. We benchmarked our modeling framework and extracted ferroelectric model parameters from experimental ferroelectric hysteresis reported in the literature, demonstrating great agreement between the model and the measurement. We further observed that ferroelectric polarization and dielectric constant decrease with increasing coercive field in general. Such an observation can provide insight for developing predictive models for ferroelectrics and optimizing ferroelectric memory.

## Results

### Preisach model

To accurately extract experimental ferroelectric hysteresis, the original computationally efficient Preisach model^27^ is modified to account for asymmetries in hysteresis loops. In the static Preisach model, the saturated hysteresis loop $P_{sat}$-$E_{FE}$ is described by Eq. (1),

1a $$\begin{aligned} P_{sat}(E_{FE})&=P_s\tanh (s\cdot (E_{FE}-E_c))+P_{offset} \end{aligned}$$

1b $$\begin{aligned} s&=\frac{1}{E_{c+}-E_{c-}}\log \bigg (\frac{P_s+P_r}{P_s-P_r}\bigg ) \end{aligned}$$

1c $$\begin{aligned} E_c&= {\left\{ \begin{array}{ll} E_{c+}, &\quad \text {if } \Delta E_{FE}>0\\ E_{c-}, &\quad \text {if } \Delta E_{FE}<0 \end{array}\right. } \end{aligned}$$

where $P_s$ is the saturation polarization, $P_r$ is the remnant polarization, *s* is the slope parameter of the $P_{FE}$-$E_{FE}$ hysteresis loop, $E_{FE}$ is the electric field in the ferroelectrics, $P_{offset}$ accounts for the shift of hysteresis loop center along the polarization axis, and $E_{c\pm }$ are the forward ($\Delta  E_{FE} > 0$) and backward ($\Delta E_{FE} < 0$) sweep coercive fields of the ferroelectric that dictate the shift of hysteresis loop center along the electric field axis, respectively. To determine the non-saturated minor hysteresis loops, a linearly scaled version of $P_{sat}$ is determined by Eq. (2)

2 $$\begin{aligned} P_{FE}(E_{FE})=m\cdot P_{sat}(E_{FE})+b \end{aligned}$$

where *m* is the proportionality factor and *b* is the offset polarization that both depend on previous hysteresis loops. Given that the turning points determined by previous hysteresis loops are ($V_{+}$, $P_{+}$) and ($V_{-}$,$P_{-}$), the coefficients *m* and *b* can be determined by Eq. (3)

3a $$\begin{aligned} m&=\frac{P_{+}-P_{-}}{P_{sat}(E_{+})-P_{sat}(E_{-})} \end{aligned}$$

3b $$\begin{aligned} b&=\frac{P_{sat}(E_{+})P_{-}-P_{sat}(E_{-})P_{+}}{P_{sat}(E_{+})-P_{sat}(E_{-})} \end{aligned}$$

3c $$\begin{aligned} E_{\pm }&= \frac{V_{\pm }}{t_{FE}} \end{aligned}$$

**Figure 2:**
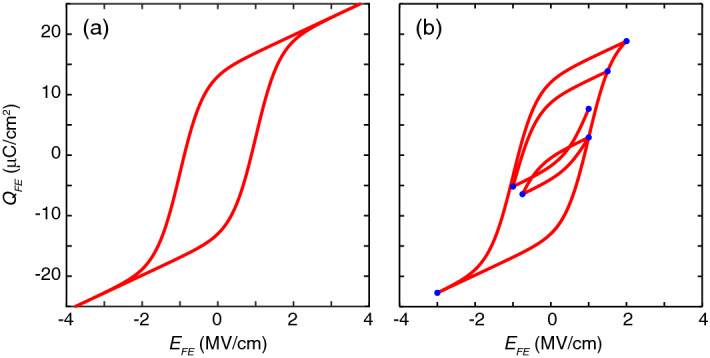
Preisach Model of hysteresis. (**a**) Simulated saturated major hysteresis loop. (**b**) Simulated non-saturated minor hysteresis loops, where the blue dots are the turning points. $P_s$ = 14 μC/cm^2^, $P_r$ = 13 μC/cm^2^, $E_{c+}$ = −  $E_{c-}$ = 1 MV/cm, $P_{offset}$ = 0 μC/cm^2^, $\varepsilon _{FE}$ = 33, and $t_{FE}$ = 10 nm are used in the simulation.

**Figure 3:**
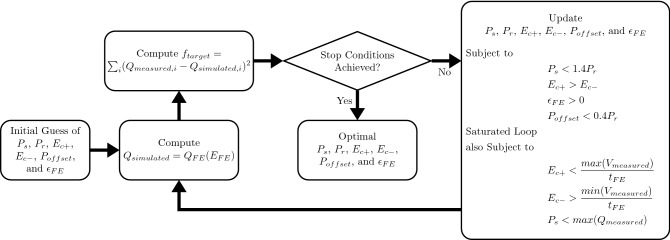
The parameter extraction procedure, where the total squared error between experimental data and simulated results is minimized using the interior-point algorithm^28^ under constraints. The optimization process stops when the desired tolerance is achieved or maximum number of iterations is reached.

The turning points ($V_{+}$, $P_{+}$) and ($V_{-}$, $P_{-}$) are the smallest possible endpoints from previous hysteresis loops that contain current hysteresis loop. If the current hysteresis loop is larger than all previous hysteresis loops, the turning points are the endpoints of the saturated major loop ($V_s$, $P_s$) and ($-V_s$, $-P_s$), where $V_s$ is much larger than $t_{FE}E_c$. Then the ferroelectric $Q_{FE}$-$E_{FE}$ relation and the saturated $Q_{FE}$-$E_{FE}$ can be obtained by summing the polarization contribution and the linear dielectric response (Eq. 4),

4a $$\begin{aligned} Q_{FE}(E_{FE})&=P_{FE}(E_{FE})+\varepsilon _0\varepsilon _{FE}E_{FE} \end{aligned}$$

4b $$\begin{aligned} Q_{sat}(E_{FE})&=P_{sat}(E_{FE})+\varepsilon _0\varepsilon _{FE}E_{FE} \end{aligned}$$

where $\varepsilon _0$ is the permittivity of free space and $\varepsilon _{FE}$ is the relative linear dielectric constant of the ferroelectrics. Simulated ferroelectric hysteresis for both saturated and non-saturated hysteresis loops are shown in Fig. 2. The parameter extraction procedure is shown in Fig. 3, where the interior-point algorithm^28^ is used to minimize the total squared error between experimental data and simulated results. Since the interior point algorithm is a local solver, a set of good initial model parameters can significantly reduce the computation time which is estimated from the measured hysteresis loops. The initial set of model parameters are generated automatically by Eq. (5).

5a $$\begin{aligned} P_{r,initial}&=0.9\max (Q_{FE}) \end{aligned}$$

5b $$\begin{aligned} P_{s,initial}&= P_r+1 \end{aligned}$$

5c $$\begin{aligned} E_{c+,initial}&=0.5\max (E_{FE}) \end{aligned}$$

5d $$\begin{aligned} E_{c-,initial}&=0.5\min (E_{FE}) \end{aligned}$$

5e $$\begin{aligned} P_{offset,initial}&=\frac{\max (Q_{FE})+\min (Q_{FE})}{2} \end{aligned}$$

5f $$\begin{aligned} \varepsilon _{FE,initial}&=\frac{1}{\varepsilon _0}\frac{dQ_{FE}}{dE_{FE}} \Big |_{\begin{array}{c} E_{FE}=\max (E_{FE}) \end{array}} \end{aligned}$$

**Figure 4:**
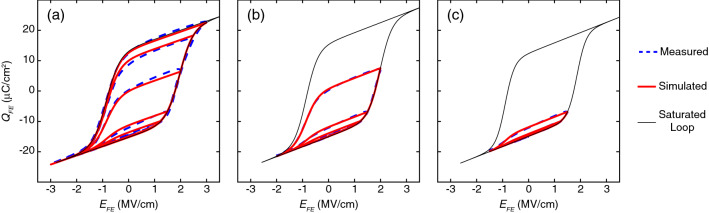
Fitted hysteresis loops based on measured data from a 10 nm HZO sample. (**a**) Fitting based on five experimental hysteresis loops, where extracted $P_s$, $P_r$, $E_{c+}$, $E_{c-}$, $P_{offset}$, and $\varepsilon _{FE}$ are 14.66 μC/cm^2^, 14.55 μC/cm^2^, 1.96 MV/cm, − 0.85 MV/cm, − 0.59 μC/cm^2^, and 33.79, respectively. (**b**) Fitting based on three experimental minor loops, where extracted $P_s$, $P_r$, $E_{c+}$, $E_{c-}$, $P_{offset}$, and $\varepsilon _{FE}$ are 15.51 μC/cm^2^, 15.45 μC/cm^2^, 1.98 MV/cm, − 0.86 MV/cm, 0.44 μC/cm^2^, and 37.15, respectively. (**c**) Fitting based on two experimental minor loops, where extracted $P_s$, $P_r$, $E_{c+}$, $E_{c-}$, $P_{offset}$, and $\varepsilon _{FE}$ are 13.71 μC/cm^2^, 13.70 μC/cm^2^, 1.86 MV/cm, − 0.90 MV/cm, − 1.19 μC/cm^2^, and 38.16, respectively. Even though the extracted model parameters vary somewhat depending on the number of hysteresis loops adopted, the reconstructed saturated loop (Eq. 4b) is much larger than the measured minor loops, alleviating the underestimated issue.

Constraints are imposed to ensure all the model parameters are reasonable as shown in the flow chart. The optimization problem is solved using the Matlab Optimization Toolbox^29^. The experimental data of a 10 nm $\hbox {Hf}_{0.5}\hbox {Zr}_{0.5}\hbox {O}_{2}$ (HZO) sample and corresponding simulated results are plotted in Fig. 4a, indicating good agreement between the model and experimental data. If only minor hysteresis loops are utilized to determine ferroelectric parameters, the extracted parameters will vary somewhat depending on the number of minor loops used. In the cases shown in Fig. 4b,c, where three and two minor loops are used for parameter extraction, respectively, the variations for $P_s$, $P_r$, and $E_c\pm$ are within 6%, the variation for $P_{offset}$ is within ±1.05 μC/cm^2^, and the variation for $\varepsilon _{FE}$ is within 13 % comparing to the case where five loops are used (Fig. 4a). Regardless of the number of minor loops used, a saturated hysteresis loop that is much larger than the minor loops is reconstructed (Fig. 4b,c). This can drastically reduce the potential underestimation of ferroelectric parameters from measured minor loops.

### Antiferroelectrics model

The aforementioned Preisach model was modified to capture the double hysteresis in antiferroelectrics through shifting. Antiferroelectricity is an electric field induce phase transition between a non-polar and a polar phase while ferroelectricity is an electric field-driven rotation of the polarization vector without a change in the crystalline symmetry. The microscopic mechanism behind these two phenomena is different, albeit related. At a mesoscopic level, both polarization rotation in ferroelectrics and the field-induced phase transition in antiferroelectrics are mediated by domain nucleation and propagation. On the other hand, the Preisach model is agnostic of the microscopic mechansim and captures only the domain dynamics occurring at the mesoscopic scale. As such, we believe that the modified Preisach model, within its limitations, captures the behavior of antiferroeletric materials as well. The saturated double hysteresis loops $P_{sat}$-$E_{FE}$ is described by Eq. (6),

6a $$\begin{aligned} P_{sat}(E_{AFE})&=P_s\tanh (s\cdot (E_{AFE}-E_{c,shift}-sgn(E_{AFE})\cdot Dir\cdot E_c))-P_{shift}+P_{offset} \end{aligned}$$

6b $$\begin{aligned} s&=\frac{1}{2E_c}\log \bigg (\frac{P_s+P_r}{P_s-P_r}\bigg ) \end{aligned}$$

6c $$\begin{aligned} P_{shift}&=P_s\tanh (s\cdot (-E_{c,shift}-sgn(E_{AFE})\cdot Dir\cdot E_c)) \end{aligned}$$

6d $$\begin{aligned} Dir&= {\left\{ \begin{array}{ll} 1, &{} \text {if } \Delta E_{AFE}>0\\ -1, &{} \text {if } \Delta E_{AFE}<0 \end{array}\right. }\end{aligned}$$

6e $$\begin{aligned}&\quad {\left\{ \begin{array}{ll} P_s=P_{s+},\quad P_r=P_{r+},\quad E_c=E_{c+},\quad E_{c,shift}=E_{c,shift+}, &\quad \text {if } E_{AFE} > 0 \\ P_s=P_{s-},\quad P_r=P_{r-},\quad E_c=E_{c-},\quad E_{c,shift}=E_{c,shift-}, &\quad \text {if } E_{AFE}<0 \end{array}\right. } \end{aligned}$$

where *s* is the slope parameter of the $P_{FE}$-$E_{AFE}$ double hysteresis loop, $E_{AFE}$ is the electric field in the antiferroelectric, *Dir* is dictated by the sweep direction of the antiferroelectric electric fields, and *sgn* is the signum function. $P_{s\pm }$ are the saturation polarization, $P_{r\pm }$ are the remnant polarization, $E_{c\pm }$ are the coercive fields, and $E_{c,shift\pm }$ are the shift in coercive fields for positive and negative $E_{AFE}$, respectively. Similarly, the non-saturated minor hysteresis loops are linearly scaled versions of $P_{sat}$ determined by Eq. (7)

7 $$\begin{aligned} P_{AFE}(E_{AFE})=m\cdot P_{sat}(E_{AFE}) \end{aligned}$$

where *m* is the proportionality factor that depends on the endpoint of the former hysteresis loop. Since antiferroelectrics are volatile, for simplicity, only one endpoint ($V_{end}$, $P_{end}$) is considered. The coefficient m can be calculated by Eq.  (8).

8a $$\begin{aligned} m&=\frac{P_{end}}{P_{sat}(E_{end})} \end{aligned}$$

8b $$\begin{aligned} E_{end}&=\frac{V_{end}}{t_{AFE}} \end{aligned}$$

**Figure 5:**
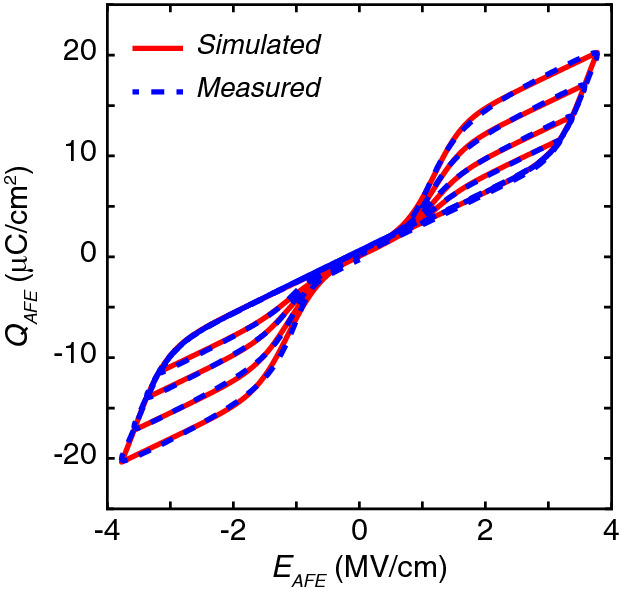
Experimental data from a 10 nm ZrO_2_ sample in excellent agreement with the antiferroelectric model for both saturated and unsaturated double hysteresis loops. The extracted $P_{s+}$, $P_{r+}$, $E_{c+}$, $E_{c,shift+}$, $P_{s-}$, $P_{r-}$, $E_{c-}$, $E_{c,shift-}$, $P_{offset}$, and $\varepsilon _{AFE}$ are 6.40 μC/cm^2^, 6.34 μC/cm^2^, 1.12 MV/cm, 2.39 MV/cm, − 6.61 μC/cm^2^, − 6.51 μC/cm^2^, − 1.22 MV/cm, − 2.35 MV/cm, 0.43 μC/cm^2^, 33.70, respectively.

Similarly, the corresponding antiferroelectric $Q_{AFE}$-$E_{AFE}$ relationship is given by Eq. (9),

9 $$\begin{aligned} Q_{AFE}(E_{AFE})=P_{AFE}(E_{AFE})+\varepsilon _0\varepsilon _{AFE}E_{AFE} \end{aligned}$$

where $\varepsilon _0$ is the permittivity of free space and $\varepsilon _{AFE}$ is the relative linear dielectric constant of the antiferroelectrics. The modified Preisach model was implemented to identify model parameters based on experimental data using the aforementioned optimization technique. Figure 5 shows excellent agreement between the experimental data from a 10 nm thick antiferroelectric ZrO_2_ sample and simulated results for both saturated major and unsaturated minor double hysteresis loops.

**Figure 6:**
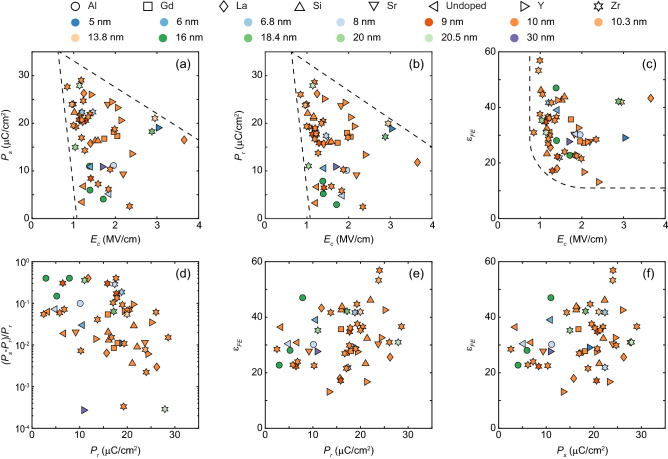
Ferroelectric parameters extracted from the literature for Al-doped^6,7^, Gd-doped^8^, La-doped^9^, Si-doped^1,10–12^, Sr-doped^13^, Y-doped^14,15^, Zr-doped^4,10,16–26^, and undoped^4,5^ HfO_2_ thin films, where $E_c$ is defined as the average of $E_{c+}$ and $|E_{c-}|$. (**a**) Saturated polarization $P_s$ vs. coercive field $E_c$. (**b**) Remnant polarization $P_r$ vs. coercive field $E_c$. (**c**) Relative linear dielectric constant $\varepsilon _{FE}$ vs. coercive field $E_c$. (**d**) ($P_s$-$P_r$)/$P_r$ vs. remnant polarization $P_r$. (**e**) Relative linear dielectric constant $\varepsilon _{FE}$ vs. remnant polarization $P_r$. (**f**) Relative linear dielectric constant $\varepsilon _{FE}$ vs. saturation polarization $P_s$.

## Discussion

**Figure 7:**
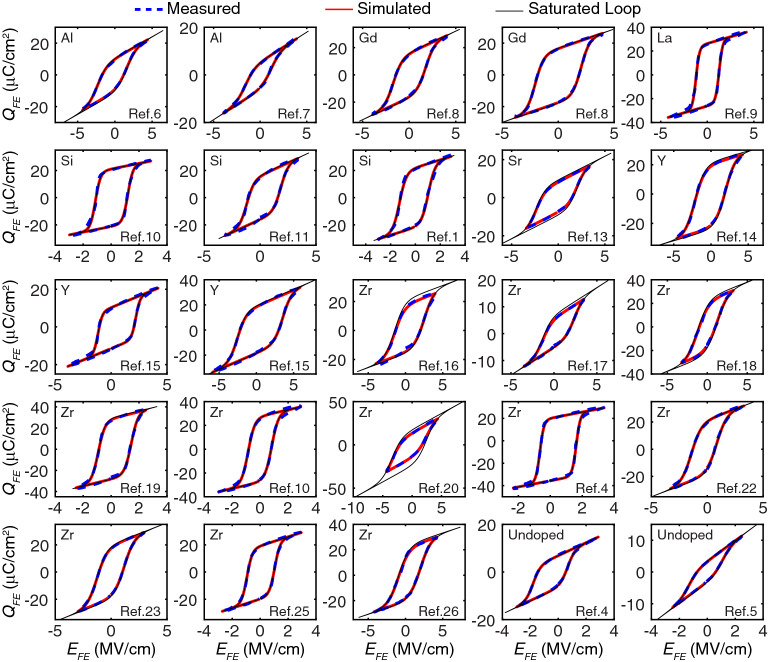
Examples of fitted hysteresis with saturated loops reconstructed based on extracted model parameters for different types of dopants.

The proposed modeling framework was used to extract ferroelectric parameters for Al-doped^6,7^, Gd-doped^8^, La-doped^9^, Si-doped^1,10–12^, Sr-doped^13^, Y-doped^14,15^, Zr-doped^4,10,16–26^, and undoped^4,5^ HfO_2_ thin films reported in the literature as shown in Fig. 6. By mapping the ferroelectric model parameters in parameter space, we ruled out unrealistic and far-fetched ferroelectric parameter combinations. We observed that as the coercive field $E_c$ increases, polarization ($P_s$ and $P_r$) and the dielectric constant $\varepsilon _{FE}$ decrease (Fig. 6a–c). The coercive field $E_c$ and dielectric constant $\varepsilon _{FE}$ show lower bounds at $\sim$1 MV/cm, and 13, respectively. Saturated polarization has an upper bound around 30 μC/cm^2^. Such systematic relation between coercive field $E_c$, polarization ($P_s$ and $P_r$), and the dielectric constant $\varepsilon _{FE}$ is interesting and suggests that there are natural limits to the material, and understanding the microscopic origin of such interdependence will require further research. To achieve large memory windows and low operation voltage in ferroelectric memory, ferroelectric materials with low dielectric constant, high polarization, and small polarization difference ($P_s$ − $P_r$) are preferred. The coercive field typically has an optimal value between 1 MV/cm and 2 MV/cm depending on other parameters because high $E_c$ hinders ferroelectric switching. Such a parametric study is crucial for ferroelectric memory design to achieve large memory windows and low program voltage under material restrictions as well as develop predictive models for ferroelectrics. Figure 7 demonstrated some fitted hysteresis loops that are used in Fig. 6. The model achieved an excellent agreement with experimental hysteresis and was able to reconstruct the saturated loops based on identified ferroelectric parameters if a non-saturated loop was measured.

## Conclusion

We developed a fast and robust modeling framework for the automated determination of ferroelectric and antiferroelectric parameters from experimental data by modifying the Preisach model. We demonstrated excellent consistency between modeled results and measured data reported from the literature. By plotting the extracted ferroelectric model parameters in parameter space, we observed that ferroelectric polarization and dielectric constant tends to decrease with the increasing coercive field. This parametric study suggests that it is important to maximize the ferroelectric polarization and minimize the dielectric constant and polarization difference ($P_s$-$P_r$) to achieve a large memory window and low voltage of operation of ferroelectric memory.

## Methods

### Sample preparation

 Both the HZO (metal-ferroelectric-metal) and ZrO_2_ (metal-antiferroelectric-metal) capacitor structures were fabricated on p+ Si (100) substrates with a native SiO_2_ layer. For the HZO sample, the bottom TiN electrode (12 nm), 10 nm $\hbox {Hf}_{0.5}\hbox {Zr}_{0.5}\hbox {O}_{2}$ film, and the top TiN electrode (12 nm) were deposited subsequently using plasma-enhanced atomic layer deposition (PEALD) technique in a Veeco Fiji G2 Plasma ALD system. The deposition was carried out at 250$^{\circ }$C using Tetrakis(dimethylamido) hafnium, zirconium, and titanium precursors with water as the oxygen source. A post-TiN metallization annealing was done on the sample at 450 $^{\circ }$C for 30 seconds in nitrogen atmosphere to crystallize the $\hbox {Hf}_{0.5}\hbox {Zr}_{0.5}\hbox {O}_{2}$ layer. For the ZrO_2_ sample, the bottom TiN layers (10 nm) and 10 nm ZrO_2_ film were deposited in a 300 mm TEL Trias$^{TM}$ clean-room tool at a temperatures of $\sim$430 $^\circ$C and 350 $^\circ$C, respectively. The temperature is high enough to crystallize ZrO_2_ during growth such that no additional annealing was required to achieve antiferroelectricity. Afterward, the top TiN layer of this sample was deposited using a Plasma Enhanced ALD (PEALD) system at Georgia Tech. After ALD and crystallization of both the samples, an Al metal layer (100 nm) patterned into rectangular shapes were evaporated to define the capacitor with areas of (200 μm)^2^, (100 μm)^2^, (50 μm)^2^, and (25 μm)^2^. The evaporated Al layer also served as a hard mask during the subsequent wet etch (1:1 H_2_O:H_2_O_2_ at 50 $^\circ$C) of the top TiN layer for patterning the top electrodes.

### Electrical characterization

 The ferroelectric sample was characterized using Keithley 4200A-SCS Parameter Analyzer using triangle waveform at 2.5 kHz, and the antiferroelectric sample was characterized using aixACCT TF Analyzer 3000 using triangle waveform at 1 kHz.

## Supplementary Information

Supplementary Information.
