# Supplementary material for: Extraction of Preisach model parameters for fluorite-structure ferroelectrics and antiferroelectrics
Source: Sci Rep. 2021 Jun 14;11:12474. doi: 10.1038/s41598-021-91492-w (PMC8203701; doi:10.1038/s41598-021-91492-w)
Supplement: Supplementary file 1 — Supplementary Information. [file 41598_2021_91492_MOESM1_ESM.pdf]

Supplementary

Extraction of Preisach model parameters for fluorite-structure ferroelectrics and antiferroelectrics

Zheng Wang[1], Jae Hur[1], Nujhat Tasneem[1], Winston Chern[2,3], Shimeng Yu[1], Asif Khan[1,4]
[1]Georgia Institute of Technology, School of Electrical and Computer Engineering, Atlanta, 30332, USA.
[2]Department of Electrical Engineering and Computer Science, Massachusetts Institute of Technology, Cambridge, MA 02142 USA.
[3]Izentis LLC, PO Box 397002, Cambridge, MA 02139, USA.
[4]Georgia Institute of Technology, School of Materials Science and Engineering, Atlanta, 30332, USA.

1. Impact of different initial parameters

To study the impact of different initial parameters, we used 4 sets of initial parameters as shown in Table 1. Set 1 is automatically generated by the program obtained by Eq.5 in the main text. Set 2-4 are scaling versions of Set 1. All 4 sets of initial parameters return the same final results, yet Set 1 use the least amount of time (Table S1). Fig.S1 compares the simulated hysteresis to the measured one.

Final result: P_s=9.31 uC/cm^2, P_r=8.92 uC/cm^2, E_{c+}=1.58 MV/cm, E_{c-}=1.70 MV/cm, P_{offset}=-0.12 uC/cm^2, ε_{FE} =29.21.

Table S1: Run time for different initial conditions.

	P_s (uC/cm^2)	P_r (uC/cm^2)	E_{c+} (MV/cm)	E_{c-} (MV/cm)	P_{offset} (uC/cm^2)	ε_{FE}	Run Time (s)
Set 1	8.8995	7.8995	2.0034	-1.9689	0	23.3032	1.34
Set 2	88.995	78.995	20.034	-19.689	10	243.032	1.65
Set 3	88.995	63.1963	10.0172	-3.9377	0	14.5819	1.96
Set 4	17.799	3.3988	6.0103	-9.8443	-10	243.032	1.57

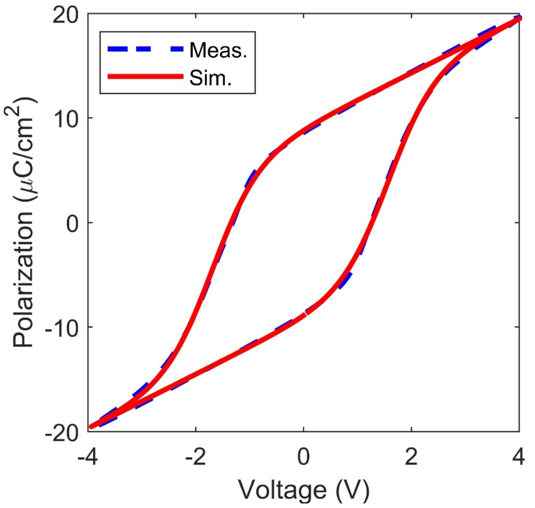

Figure S1: Comparison between the simulation and the measurement

2. Comparison of parameters from different extraction methods

We compared the parameters extracted using the modeling framework proposed in this work to the parameters extracted using direct determination for the hysteresis loop shown in Fig.S1.

Table S2: Comparison of parameters from different extraction methods

	P_s (uC/cm^2)	P_r (uC/cm^2)	E_{c+} (MV/cm)	E_{c-} (MV/cm)	P_{offset} (uC/cm^2)	ε_{FE}
Direct Determination	N/A (10.51)	8.72	1.25	1.30	-0.056	N/A (25.68)
This work	9.31	8.92	1.58	1.70	-0.12	29.21
Error (%)	N/A (12.9)	-2.2	-20.9	-23.5	53.3	N/A (-12.1)

Ps and ε_{FE} is not obvious in a ferroelectric hysteresis. Typically, PUND and CV measurements are performed to extract these parameters. However, approximations can be made to get rough numbers for these two parameters. ε_{FE} can be approximated by the slope of the hysteresis loop near the end points (Eq.S1).

$$\epsilon_{FE} = \frac{1}{\epsilon_0}\frac{dQ_{FE}}{dE_{FE}}\Big|_{E_{FE}=\max(E_{FE})} \tag{S1}$$

Ps can be approximated by Eq.S2

$$P_s = \frac{1}{2}\left(\max(Q_{FE}) - \min(Q_{FE}) - \epsilon_0\epsilon_{FE}(\max(E_{FE}) - \min(E_{FE}))\right) \tag{S2}$$

The approximated values for Ps and ε_{FE} are shown in the parenthesis in Table S2. Notice that P_{offset} is small and hence prone to have large percentage error. We observed that $E_{c\pm}$ shows large error because direct determination method evaluates $E_{c\pm}$ of a Q_{FE}-E_{FE} hysteresis (Eq.S3a) rather than a P_{FE}-E_{FE} hysteresis (Eq.S3b).

$$Q_{FE} = P_s \tanh\big(s \cdot (E_{FE} - E_c)\big) + P_{offset} + \epsilon_0 \epsilon_{FE} E_{FE} = 0 \qquad \text{(S3a)}$$

$$P_{FE} = P_s \tanh\big(s \cdot (E_{FE} - E_c)\big) + P_{offset} = 0 \qquad \text{(S3b)}$$

For simplicity, we assume P_{offset} = 0 uC/cm^2 and $E_c = E_{c+} = -E_{c-}$. Since Eq.S3a cannot be solved analytically, we expand it in Taylor series at $E_{FE} = E_c$.

$$Q_{FE} = (P_s s + \epsilon_0 \epsilon_{FE})(E_{FE} - E_c) + \epsilon_0 \epsilon_{FE} E_c = 0 \qquad \text{(S4a)}$$

$$E_{c,ext} = E_{FE} = \frac{s E_c P_s}{P_s s + \epsilon_0 \epsilon_{FE}} = \frac{E_c}{1 + \frac{\epsilon_0 \epsilon_{FE}}{P_s s}} \qquad \text{(S4b)}$$

Eq.S3b yields:

$$E_{c,int} = E_{FE} = E_c \qquad \text{(S4c)}$$

Evaluate Eq.S4b with parameters extracted based on the method proposed in this work,

$$s = \frac{1}{2E_c} \log\left(\frac{P_s + P_r}{P_s - P_r}\right) = 1.217 \qquad \text{(S5a)}$$

$$E_{c,ext} = \frac{E_c}{1 + \frac{\epsilon_0 \epsilon_{FE}}{P_s s}} = 0.81 E_c \qquad \text{(S5b)}$$

Comparing Eq.S4c and Eq.S5b, we noticed that the direct determination method induced ~20% error for E_c in this case.

3. Convergence behavior of the parameter extraction process

Fig.S2 shows the error as a function of number of iterations during the extraction process for the hysteresis shown in Fig.S1. The target function (error) decreases monotonically and converges to a constant. Fig.S3-S7 show the evolution of the simulated hysteresis at iteration 1, 2, 5, 10, and 20, respectively.

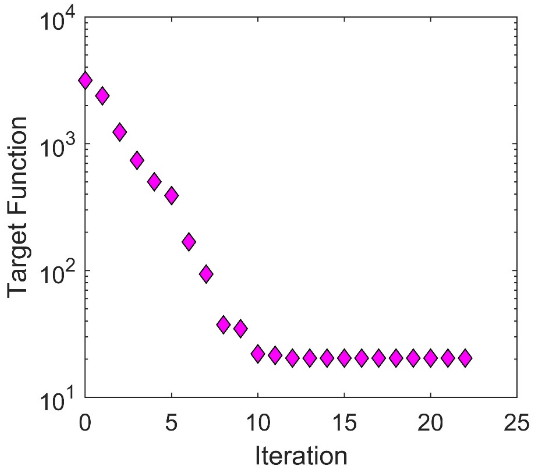

Figure S2: Error vs. iteration for the hysteresis shown in Fig.S1

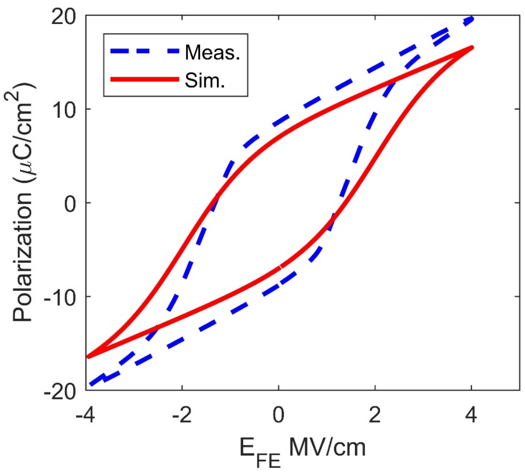

Figure S3: Simulated hysteresis at Iteration 1

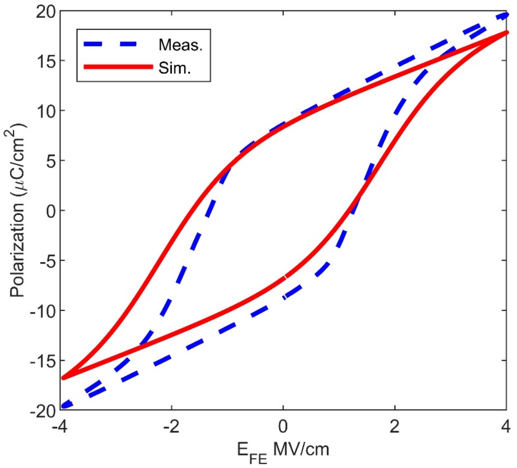

Figure S4: Simulated hysteresis at Iteration 2

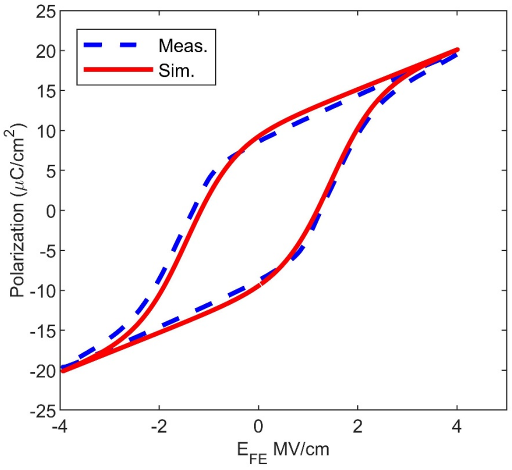

Figure S5: Simulated hysteresis at Iteration 5

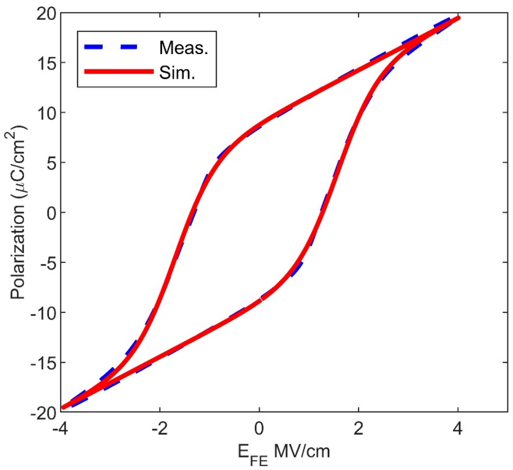

Figure S6: Simulated hysteresis at Iteration 10

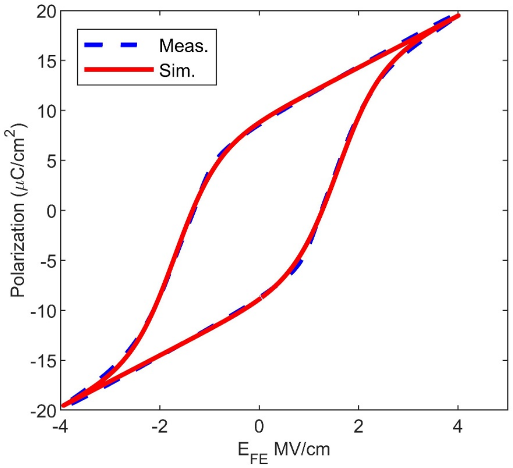

Figure S7: Simulated hysteresis at Iteration 20

Fig. S8-S10 show the convergence plot for Fig.4 in the main text where different number of minor loops are used for parameter extraction.

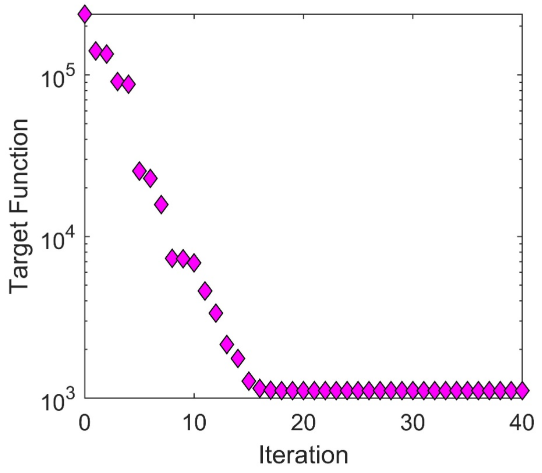

Figure S8: Error vs. iteration for the hysteresis shown in Fig 4(a) in the main text where 5 minor loops are used

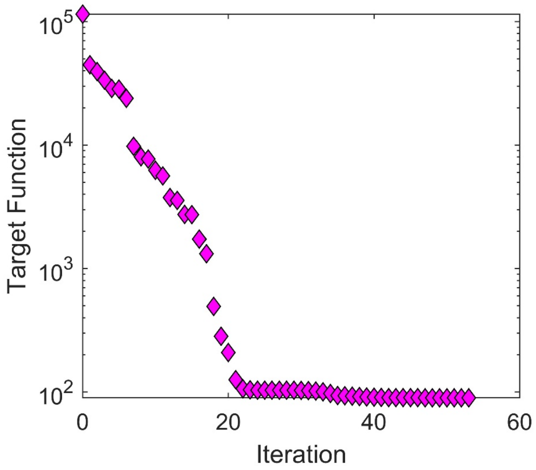

Figure S9: Error vs. iteration for the hysteresis shown in Fig 4(b) in the main text where 3 minor loops are used

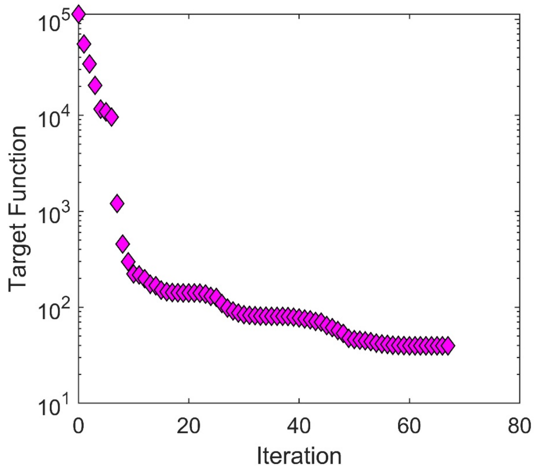

Figure S10: Error vs. iteration for the hysteresis shown in Fig 4(b) in the main text where 2 minor loops are used
